# Deranged Bioenergetics and Defective Redox Capacity in T Lymphocytes and Neutrophils Are Related to Cellular Dysfunction and Increased Oxidative Stress in Patients with Active Systemic Lupus Erythematosus

**DOI:** 10.1155/2012/548516

**Published:** 2011-10-11

**Authors:** Ko-Jen Li, Cheng-Han Wu, Song-Chou Hsieh, Ming-Chi Lu, Chang-Youh Tsai, Chia-Li Yu

**Affiliations:** ^1^Department of Internal Medicine, National Taiwan University Hospital and National Taiwan University College of Medicine, 7 Chung-Shan South Road, Taipei 100, Taiwan; ^2^Institute of Clinical Medicine, National Yang-Ming University School of Medicine, 109, Section 2, Li-Nong Street, Taipei 112, Taiwan; ^3^Division of Immunology, Rheumatology, and Allergy, Buddhist Dalin Tzu-Chi General Hospital, 2 Ming-Shen Road, Chia-Yi 622, Taiwan; ^4^Division of Allergy, Immunology, and Rheumatology, Taipei Veterans General Hospital, 201 Shih-Pai Road, Taipei 112, Taiwan

## Abstract

Urinary excretion of *N*-benzoyl-glycyl-*N*ε**-(hexanonyl)lysine, a biomarker of oxidative stress, was higher in 26 patients with active systemic lupus erythematosus (SLE) than in 11 non-SLE patients with connective tissue diseases and in 14 healthy volunteers. We hypothesized that increased oxidative stress in active SLE might be attributable to deranged bioenergetics, defective reduction-oxidation (redox) capacity, or other factors. We demonstrated that, compared to normal cells, T lymphocytes (T) and polymorphonuclear neutrophils (PMN) of active SLE showed defective expression of facilitative glucose transporters GLUT-3 and GLUT-6, which led to increased intracellular basal lactate and decreased ATP production. In addition, the redox capacity, including intracellular GSH levels and the enzyme activity of glutathione peroxidase (GSH-Px) and **γ**-glutamyl-transpeptidase (GGT), was decreased in SLE-T. Compared to normal cells, SLE-PMN showed decreased intracellular GSH levels, and GGT enzyme activity was found in SLE-PMN and enhanced expression of CD53, a coprecipitating molecule for GGT. We conclude that deranged cellular bioenergetics and defective redox capacity in T and PMN are responsible for cellular immune dysfunction and are related to increased oxidative stress in active SLE patients.

## 1. Introduction

Systemic lupus erythematosus (SLE) is an archetype of systemic autoimmune disease that is characterized by diverse immune dysfunctions. SLE patients experience increased oxidative stress that is related to mitochondrial hyperpolarization and ATP depletion [1–4]. However, the molecular basis of this increased oxidative stress and its relationship to immune dysfunction in SLE patients remain unclear. Active immune cells such as lymphocytes and polymorphonuclear neutrophils (PMN) need constant energy for both basic housekeeping and specific immune functions such as tissue immigration, antigen processing/presentation, signal transduction, and other effector functions [5, 6]. The oxidation of glucose provides a major source of metabolic energy in mammalian cells. Glucose-6-phosphatase is an enzyme that is only present in mammalian liver and kidneys and plays an important role in producing glucose during periods of starvation [7]. Because the lipid bilayer on the mammalian cell surface is impermeable to the hydrophilic and polar glucose molecules, cellular uptake of glucose is only achieved through glucose carriers embedded in the bilayer. The glucose transporters include sodium-dependent cotransporters and facilitative glucose transporters (GLUT) that facilitate glucose diffusion along a concentration gradient [8]. Each of the 14 isoforms of facilitative glucose transporters (GLUT-1 to GLUT-13 and HMIT-1), exhibits a different affinity for glucose and other hexoses. GLUT-1 and GLUT-3 possess a high affinity for glucose [9]. GLUT-1 is present at variable concentrations in many tissues and is believed to be responsible for basal glucose uptake [10]. GLUT-3 is mainly expressed in brain and circulating mononuclear cells [11]. GLUT-6 is only expressed in the spleen, leukocytes, and brain [12, 13]. There have been few studies on the relationship between glucose transporter expression and the bioenergetics of SLE immune-related cells reported in the literature.

Viora et al. [14] demonstrated that the intracellular reduction-oxidation (redox) state might affect lymphocyte proliferation and NK-mediated cytotoxicity. The glutaredoxin-glutathione system is the key player in redox regulation of the cells and is composed of NADPH, reduced-form glutathione (GSH), the flavoprotein glutathione peroxidase (GSH-Px), and glutathione reductase (GSSG-Rx) [15]. Because glutathione is present in all animal cells in high concentrations, it acts as the most important intracellular modulator for redox, cell proliferation, DNA synthesis, immune responses, and arachidonic acid metabolism [16, 17]. The antioxidant glutathione must be recaptured by *γ*-glutamyl-transpeptidase (GGT) [18]. Because CD53, a glycoprotein of the tetraspanin superfamily, can coprecipitate with GGT activity, elevated CD53 expression in an enhanced oxidative stress environment may prevent cell apoptosis due to oxidative damage [19]. In the present study, we hypothesized that deranged bioenergetics and defective redox capacity, especially in the immune-active cells, may be involved in the molecular basis of cellular immune dysfunction and increased oxidative stress in patients with active SLE. Our results support this hypothesis. 

## 2. Patients and Methods

### 2.1. Patients and Controls

Fifty-four patients meeting the 2000 ACR revised classification criteria for SLE were enrolled in the study. An additional 52 age- and sex-matched healthy individuals comprised the normal control group. A second control group comprised of patients with non-SLE connective tissue diseases was created to enable us to compare the urinary excretion of *Nε*-HEL and 8-OHdG of such patients to that of SLE patients. The 11 patients in the second control group included patients with vasculitis (*n* = 2), Sjogren's syndrome (*n* = 3), rheumatoid arthritis (*n* = 4), antiphospholipid syndrome (*n* = 1), and systemic sclerosis (*n* = 1). Patients with SLE were divided into two groups. Forty-two patients with an SLEDAI score ≥6 were considered to have active SLE [20]. Twelve patients with scant clinical manifestations and who had normal levels of C3/C4 and anti-dsDNA were placed in the inactive SLE group. The glucose uptake of immune cells in the inactive SLE group was compared to that of cells from the active SLE group. The demographic, laboratory, and clinical data of all three groups are listed in Table 1. This study was approved by the Institutional Review Board and Ethical Committee, National Taiwan University Hospital, Taipei, Taiwan. Informed consent was obtained from each participant. Venous blood and 24-hour urine were collected from each participant for analysis.

### 2.2. Detection of 24-Hour Excretion of N-Benzoyl-Glycyl-N*ε*-(Hexanonyl)Lysine (N*ε*-HEL) and 8-Hydroxy-2-Deoxyguanosine (8-OHdG)

We collected all urine excreted over a 24-hour period from 26 active SLE, 11 non-SLE, and 14 healthy volunteers in clean containers. Urinary *Nε*-HEL and 8-OHdG were quantified using commercially available kits (Japan Institute for Aging, Schizuoka, Japan). The concentration of urine creatinine (Ucre) was concomitantly measured. The excretion of *Nε*-HEL was calculated as pmol/mg Ucre, and 8-OHdG was calculated as pg/mg Ucre. 

### 2.3. Isolation of T Lymphocytes and Polymorphonuclear Neutrophils from Peripheral Blood

Heparinized venous blood obtained from participants was mixed with one-quarter volume of 2% dextran solution (molecular weight, 464,000 daltons; Sigma-Aldrich Company, St. Louis, MO, USA) and incubated at 37°C for 20 min. The leukocyte-rich supernatant was collected and diluted with the same volume of Hanks' balanced salt solution. The cell suspension was layered on a Ficoll-Hypaque density gradient cushion (specific gravity 1.077, Pharmacia Biotech, Uppsala, Sweden) and centrifuged at 150 g for 30 min. The mononuclear cells (MNC) were aspirated from the interface and PMN were collected from the bottom. The RBC in the PMN suspension were lysed by incubation in 0.83% ammonium chloride solution chilled to 4°C for 10 min. To purify the T lymphocyte samples, the MNC suspension was positively selected by monoclonal antihuman CD3 antibody-coated microbeads and AutoMACS (Miltenyi Biotec, Bergisch, Gladback, Germany), as in our previous report [21]. PMN and T lymphocytes were more than 95% viable and pure, as confirmed by trypan blue dye exclusion and analysis with flow cytometry after staining with FITC-conjugated anti-CD16 (for PMN) or anti-CD3 (for T cell) antibody (Sigma-Aldrich).

### 2.4. Measurement of Intracellular Basal Lactate Levels in T and PMN

Intracellular basal lactate was measured using the method reported by Frauwirth et al. [22]. Briefly, we used enzymatic diagnostic kits (Sigma-Aldrich) to measure the intracellular basal lactate levels (mg/dL) in cell lysates (2 × 10^6^ cells/mL) of T cells and PMN from normal and SLE patients. A detailed description of the procedures can be found in the manufacturer's instruction booklet.

### 2.5. Measurement of Intracellular ATP Levels in T and PMN

Intracellular ATP levels (nmole/10^6^ cells/mL) in T and PMN lysates were determined by using ATP determination kits (Molecular Probes, Eugene, OG, USA). Detailed procedures are described in the manufacturer's instruction booklet. 

### 2.6. Measurement of Spontaneous Glucose Uptake and Glucose Transporter Expression on T and PMN

**(a) Measurement of Spontaneous Glucose Uptake of the Cells by ^3^H-2-Deoxy-d-Glucose Incorporation Assay**

Spontaneous glucose uptake of T and PMN was measured using the method described by Shikhman et al. [23]. Briefly, PMN (1 × 10^7^ cells/mL) were incubated with 10 *μ*L of radio-labeled ^3^H-deoxy-d-glucose (specific activity, 15–25 Ci/mL; Roche Diagnostics, Indianapolis, IN, USA) at room temperature for 7 min. The cells were washed three times with cold PBS and then lysed with Cell Death Lysis buffer (Sigma-Aldrich). The radioactivity of the cells was detected by a *β*-counter.

**(b) Measurement of Spontaneous Expression of Glucose Transporters, GLUT3, and GLUT6 on T and PMN by Flow Cytometry**

The facilitative glucose transporters GLUT-3 and GLUT-6 are differentially expressed in different mammalian blood cells and brain tissues [11]. The spontaneous expression of GLUT-3 and GLUT-6 glucose transporters on T and PMN was ascertained by first staining with FITC-labeled monoclonal antibody against human GLUT-3 or GLUT-6 (Chemicon Company, Temecula, CA, USA) followed by FACSort flow cytometric analysis with 488 nm excitation (Becton-Dickenson, Franklin Lakes, NJ, USA).

### 2.7. Measurement of Intracellular GSH Concentration in T and PMN

Soluble cellular GSH concentration was measured using BIOXYTECH GSH-400 colorimetric assay kit (OXIS International Inc., Portland, OR, USA). Detailed procedures are provided in the manufacturer's instruction booklet. Briefly, the concentration of T and PMN in test samples was adjusted to 1 × 10^7^ cells/mL and sonicated at 100 W for 60 s. Only the soluble cellular forms, GSH and glutathione disulfide, were detected by the kit. The detection limit of the assay is 0.5 *μ*M/mL.

### 2.8. Determination of GSH-Px Enzyme Activity

We used BIOXYTECH GPx-340 colorimetric assay kits (OXIS International Inc.) to measure the GSH-Px enzymatic activity of T and PMN cell lysates. One milliunit (mU) of GSH-Px activity is defined as the activity that catalyzes the oxidation of 1 nmol NADPH/mL/min, using an extinction molar coefficient of 6.22 × 10^6^ M^−1^·mL^−1^ for NADPH. 

### 2.9. Determination of GSSG-R Activity

We used BIOXYTECH GR-340 colorimetric assay kits (OXIS International Inc.) to measure the GSSG-R enzyme activity of T and PMN cell lysates. The definition of 1 mU of GSSG-R enzymatic activity is the activity that catalyzes the reduction of 1 nmol NADP^+^/mL/min.

### 2.10. Detection of GSH-Px mRNA Expression in T and PMN by RT-PCR

Total cellular RNA was extracted from 1 × 10^7^/mL of T or PMN using an Ultraspec RNA isolation kit (Biotex Laboratories, Houston, TX, USA). Each extracted sample (5 *μ*g) was reversely transcribed into cDNA by placement in 30 *μ*L of reverse transcriptional buffer for 1 h at 42°C. The buffer contained 50 mM Tris-HCl, 75 mM KCl, 3 mM MgCl_2_, 0.5 mg oligo-dT primer, 0.5 mM dNTP, 32 U RNasin, 10 mM DTT, and 40 U MMLV reverse transcriptase (Promega, Madison, WI, USA) at pH 8.3. The reverse transcription products (5 *μ*L) were added to a PCR buffer containing 10 mM Tris-HCl, 1.5 mM MgCl_2_, 50 mM KCl, 0.1% Triton X-100, 100 ng forward primer, 100 ng reverse primer, 0.2 mM dTNP, 2 U DNA polymerase (Promega), and 5% DMSO. PCR was performed in a Hybaid OmniGene DNA thermocycler (Teddington, UK) with a program of denaturing at 95°C for 1 min, annealing at 50–58°C for 1 min, and primer extension at 72°C for 1 min. The amplification was carried out for 25–35 cycles. The reaction was stopped after a final extension at 72°C for 10 min followed by incubation at 25°C. The forward and reverse pair primers for human GSH-Px and glyceraldehyde-3-phosphate dehydrogenase (G3PDH, used as an internal control) are shown below:

GSH-Px: 5′-GGG GCC TGG TGG TGC TCG GCT-3′ (sense),
5′-CAA TGG TCT GGA AGC GGC GGC-3′ (antisense),
G3PDH: 5′-ACC ACA GTC CAT GCC ATC AC-3′ (sense),
5′-TCC ACC ACC CTG TTG CTG TA-3′ (antisense).

The amplified PCR products were 354 bp for GSH-Px and 452 bp for G3PDH. 

### 2.11. Western Blot Analysis of GSH-Px Isomers

T and PMN at a concentration of 5 × 10^6^/mL were lysed and electrophoresed in 10% SDS-PAGE. The distribution of GSH-Px isomers in cell lysates was detected by monoclonal antihuman GSH-Px antibody (MBL International, Woburn, MA, USA) and enhanced chemiluminescence protein detection kits (Amersham International Plc., Chalfont, Buckinghamshire, UK) after electrotransfer to a nitrocellulose membrane.

### 2.12. Determination of GGT Enzymatic Activity in T and PMN

We followed the method outlined by Carlisle et al. to quantify GGT activity in T and PMN [18]. Briefly, 1 × 10^6^ cells were suspended in 1 mL of PBS containing 2.5 mM *γ*-glutamyl-*p*-nitroanilide and 60 mM glycyl-glycine at pH 7.2. After 90 min of incubation at 37°C, the cells were centrifuged and the absorbance of the supernatant was read at OD_410_ nm. The enzyme activity was calculated from the absorbance readings, and is expressed as *μ*mol *p*-nitroaniline released/min/10^6^ cells.

### 2.13. Detection of Surface-Expressed CD53 on T and PMN

The direct immunofluorescence antibody method, as reported by Pedersen-Lane et al., was employed to stain the CD53 surface expression on T and PMN [19]. We used FITC-labeled mouse monoclonal antibody against human CD53 purchase from BD Biosciences (San Jose, CA, USA) for this assay.

### 2.14. Statistical Analysis

All results are presented as mean ± S.D. The statistical significance of differences between groups was assessed by nonparametric Wilcoxon rank-sum tests using the commercially available software package: Stata/SE 8.0 for Windows. A *P* value ≤0.05 was considered to be statistically significant. 

## 3. Results

### 3.1. Increased Oxidative Stress in Active SLE Patients Detected by Surrogate Urinary Biomarkers, N*ε*-HEL, and 8-OHdG Excretion

**Figure 1:**
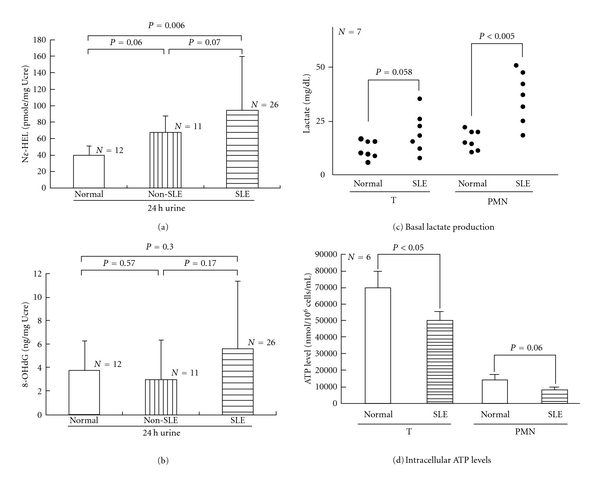
Comparison of 24-hour urinary excretion of *N*-benzoyl-glycyl-*Nε*-(hexanoyl)lysine (*Nε*-HEL) and 8-hydroxy-2-deoxyguanosine (8-OHdG), intracellular basal lactate levels, and ATP production of T lymphocytes and PMN from normal individuals, non-SLE patients, and patients with active SLE. (a) Urinary *Nε*-HEL excretion denoted by pmole/mg urine creatinine (Ucre). (b) Urinary 8-OHdG excretion denoted by ng/mg Ucre. (c) Intracellular basal lactate levels. (d) ATP production.

Oxidative stress can induce peroxidation of polyunsaturated fatty acid resulting in the production of *Nε*-HEL [24]. In addition, C-8 in the guanine residue of DNA is easily cleaved by hydroxyl free radicals. The production of 8-OHdG is the hydroxidative product of guanine after free radical hydroxylation [25]. Both *Nε*-HEL and 8-OHdG are excreted in the urine, and we used these oxidative products as biomarkers for oxidative stress. We measured the 24-hour urinary excretion of *Nε*-HEL, 8-OHdG and creatinine (Ucre) concomitantly. We found *Nε*-HEL secretion in active SLE patients was significantly higher than in non-SLE and normal individuals (Figure 1(a)). However, the excretion of 8-OHdG did not differ between the 3 groups (Figure 1(b)). These results suggest that *Nε*-HEL excretion is increased in active SLE and is consistent with the findings of other authors [1, 4]. Whether *Nε*-HEL urinary excretion in the non-SLE group is abnormal cannot be determined from our data due to the variety of diseases and the small sample size.

### 3.2. Deranged Cellular Bioenergetics with Increased Intracellular Basal Lactate Levels and Decreased ATP Production in SLE T Lymphocytes and PMN

We hypothesized that both deranged bioenergetics and defective redox capacity may attribute to increased oxidative stress in patients with active SLE. Accordingly, we measured the intracellular basal lactate levels and intracellular ATP production as indicators of cellular bioenergetics. As demonstrated in Figure 1(c), intracellular basal lactate levels in both SLE-T and SLE-PMN were higher than in normal cells. The ATP production in both T and PMN of SLE patients was below normal (Figure 1(d)). This may due to mitochondrial hyperpolarization in the preexcited immune cells of active SLE *in vivo* [3, 26]. Our results suggest that defective cellular bioenergetics of the immune active cells is one of the causes of increased oxidative stress in patients with active SLE.

### 3.3. Decreased Glucose Uptake and GLUT-3, and GLUT-6 Expression on T and PMN of SLE Patients

**Figure 2:**
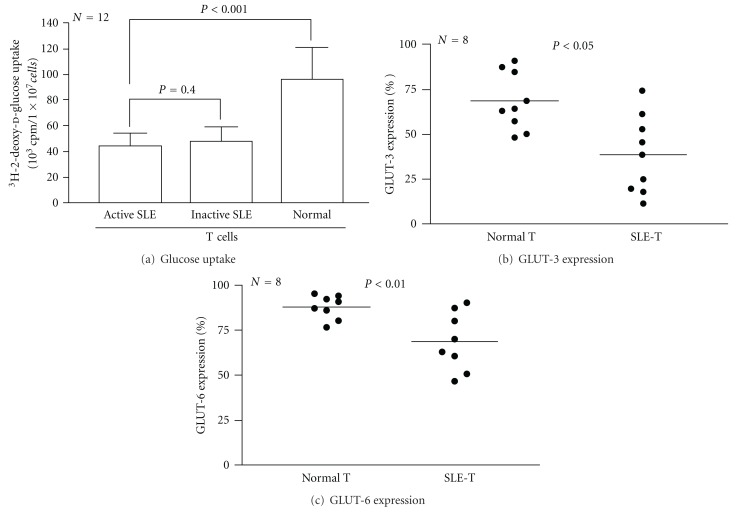
Comparison of glucose uptake and expression of glucose transporter type 3 (GLUT-3) and type 6 (GLUT-6) on T lymphocytes in normal and active SLE groups. (a) Glucose uptake by the cells detected by ^3^H-2-deoxy-d-glucose incorporation after 24-hour incubation. (b) Expression of GLUT-3 on normal and active SLE T lymphocytes. (c) Expression of GLUT-6 on normal and active SLE T lymphocytes.

**Figure 3:**
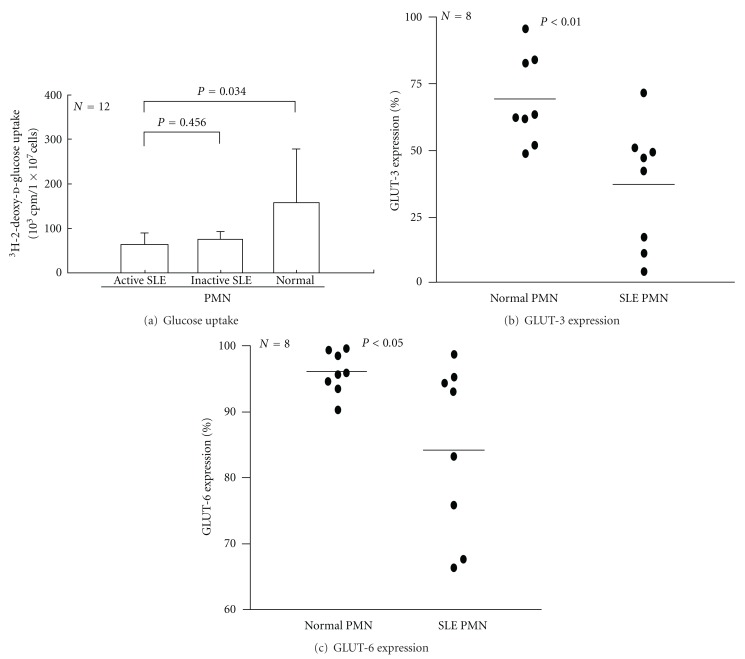
Comparison between normal and SLE-PMN glucose uptake and expression of glucose transporter type 3 (GLUT-3) and type 6 (GLUT-6). (a) Glucose uptake of the cells was detected by ^3^H-2-deoxy-d-glucose incorporation after 24-hour incubation. (b) Expression of GLUT-3 on normal and active SLE-PMN. (c) Expression of GLUT-6 on normal and active SLE-PMN.

To determine whether the abnormal cellular bioenergetics of SLE immune active cells is related to decreased glucose uptake leading to high rate of lactate production and a subsequent induction of proapoptotic *Bcl-2* gene expression even under aerobic conditions [27–29], we detected cellular glucose-uptake by ^3^H-2-deoxy-d-glucose incorporation. As shown in Figure 2(a) (T lymphocytes) and Figure 3(a) (PMN), the glucose uptake by SLE cells was significantly lower than normal cells regardless of active or inactive SLE status. These results suggest that decreased expression of facilitative glucose transporters GLUT-3 and GLUT-6 is congenital rather than acquired in SLE patients. This defect may contribute to deranged cellular bioenergetics in SLE patients. 

### 3.4. Comparison of Redox Capacity in Plasma and Different Normal Blood Cell Populations

**Figure 4:**
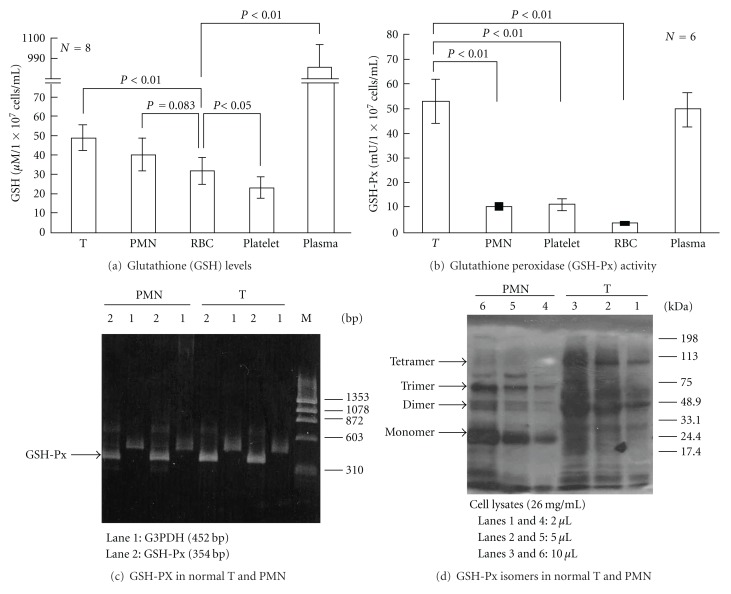
Comparison of intracellular reduced-form glutathione (GSH) levels, enzyme activity, and gene expression of glutathione peroxidase (GSH-Px) in the plasma and different blood cell populations of normal individuals. (a) Intracellular GSH levels (*μ*M/1 × 10^7^ cells/mL) in T, PMN, red blood cells, platelets, and plasma of normal individuals. (b) GSH-Px enzyme activity (mU/1 × 10^7^ cells/mL) in plasma and different blood cells of normal individuals. One milliunit (mU) of GSH-Px enzyme activity is the activity that catalyzes the oxidation of 1 nmol NADPH/mL/min. (c) Expression of GSH-Px mRNA in T and PMN of two normal individuals by RT-PCR, lane 1: G3PDH (452 bp, as internal control), lane 2: GSH-Px (354 bp). (d) A representative case demonstrating dose-response expression of GSH-Px isomers in a normal T and a normal PMN by Western blot. Three doses (2 *μ*L in lanes 1 and 4; 4 *μ*L in lanes 2 and 5; 10 *μ*L in lanes 3 and 6) of cell lysates (protein concentration 26 mg/mL) were analyzed in Western blot probed by antihuman GSH-Px antibody. Four GSH-Px isomers are identified as monomer (25 kDa), dimer (50 kDa), trimer (75 kDa), and tetramer (100 kDa). Two normal samples exhibited a similar tendency.

Cellular redox capacity is reduced in active SLE and is a major factor contributing to oxidative stress. Reduced-form glutathione is one of the most important endogenous molecules for modulating the redox state of all animal cells [15–17]. We measured the plasma GSH levels and intracellular GSH levels in different blood cell populations including T cells, PMN, RBC, and platelets of normal individuals (Figure 4). GSH is most abundant in the plasma and T lymphocytes and GSH levels of PMN > RBC > platelets (Figure 4(a)). The reduced form GSH is generated by the activity of GSH-Px on the oxidized form, GSSG [1]. GSH-Px enzymatic activity was higher in T and plasma than in PMN, RBC, and platelets, paralleling GSH levels in the normal blood subpopulations (Figure 4(b)). Although the total expression of GSH-Px mRNA in normal T and PMN did not differ in 2 cases shown in Figure 4(c), the composition and distribution of GSH-Px isomers in normal T and normal PMN are quite different, as determined by 10% SDS-PAGE analysis. As shown in Figure 4(d), T lymphocytes expressed mainly dimer (50 kDa) and tetramer (100 kDa) isomers rather than monomer (25 kDa) and trimer (75 kDa) isomers of GSH-Px. In contrast, PMN expressed mainly monomer and trimer isomers, rather than dimer and tetramer isomers, of GSH-Px. These results are compatible with those of Misso et al. [30], who showed that normal neutrophils contain mainly monomer and trimer isoforms of GSH-Px. Because the GSH-Px enzymatic activity of T lymphocytes is much higher than that of PMN, we speculated that the dimer and tetramer isoforms of GSH-Px may possess more potent antioxidant activity than the monomer and trimer isoforms. 

### 3.5. Defective Redox Capacity (GSH Levels, GSH-Px, and GSSG-R Activity) in Plasma, T, and PMN of SLE Patients

**Figure 5:**
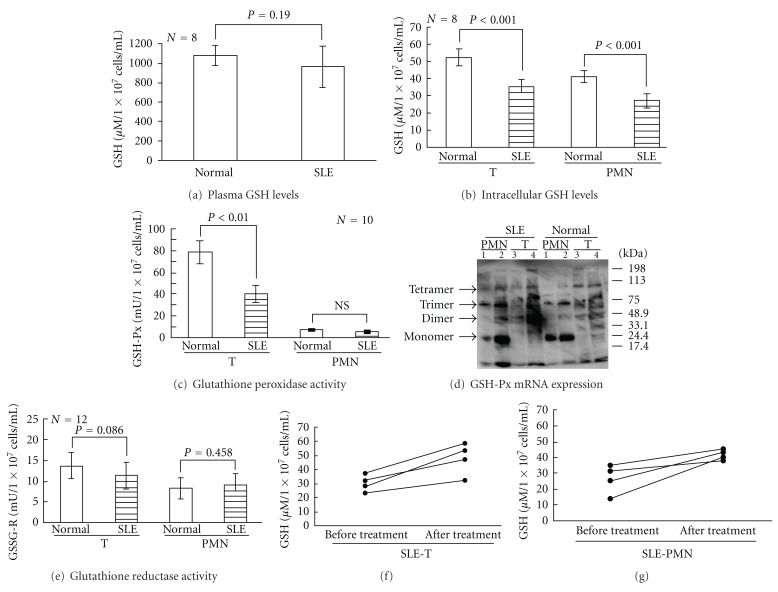
Comparison of plasma and intracellular GSH levels, glutathione peroxidase (GSH-Px) enzyme activity, GSH-Px isomer expression, and glutathione reductase (GSSG-R) enzyme activity in T and PMN from normal and active SLE groups. (a) Plasma GSH levels. (b) Intracellular GSH levels (*μ*M/1 × 10^7^ cells/mL) in T and PMN of normal and SLE patients. (c) GSH-Px (mU/1 × 10^7^ cells/mL) enzyme activity in T and PMN of normal and active SLE group. One milliunit (mU) of GSH-Px enzyme activity is the activity that catalyzes the reduction of 1 nmol NADP^+^/mL/min. (d) Western blot analysis of GSH-Px isomer distribution in two cases of T and PMN from 2 normal and 2 SLE patients. Both normal PMN and SLE-PMN contain mainly monomer (25 kDa) and trimer (75 kDa) isomers rather than dimer (50 kDa) and tetramer (100 kDa) isomers. In contrast, dimer (50 kDa) and tetramer (100 kDa) isomers were the main isomers found in normal and SLE-T cells. Lanes 1 and 2 are different cases of PMN. Lanes 3 and 4 are different cases of T. (e) Comparison of GSSG-R enzyme activity in T and PMN of normal and SLE group members. (f) Intracellular GSH levels in active SLE-T before and after effective treatment. (g) Intracellular GSH levels in active SLE-PMN before and after effective treatment.

The plasma GSH levels in SLE patients was not different from that of normal individuals (Figure 5(a)). However, the GSH levels in SLE-T and SLE-PMN (Figure 5(b)), and the GSH-Px enzymatic activity in SLE-T were significantly lower than their normal counterparts (Figure 5(c)). The very low GSH-Px enzyme activity in PMN made it difficult to detect any differences between SLE and normal groups (Figure 5(c)). Unexpectedly, the distribution of the four GSH-Px isomers in SLE-T and SLE-PMN cells was not different from that of their normal counterparts (Figure 5(d)). The activity of GSSG-R, a redox-modulating enzyme that contains active dithiol moieties for protection and repair of protein sulfhydryls in oxidative stress situations [1], was not different between normal individuals and SLE patients (Figure 5(e)). To determine whether immunosuppressants such as glucocorticoids, hydroxyl-chloroquine, and azathioprine, or SLE disease activity *per se* affects the decreased redox capacity observed in SLE patients, we determined the redox capacity of T and PMN in 4 active SLE patients before and after effective treatment. We found that intracellular GSH levels in T and PMN of nontreated active SLE patients were defective in a manner similar to that of immunosuppressant-treated active SLE patients, but the low intracellular GSH levels in active SLE patients recovered after the immunosuppressant treatment were effective (Figures 5(f) and 5(g)). These results suggest that the reduced redox capacity of active SLE-T and SLE-PMN originates from lupus disease activity, rather than the effects of immunosuppressants. 

### 3.6. Comparison of GGT Activity and CD53 Expression on T and PMN of Normal and SLE Patients

**Figure 6:**
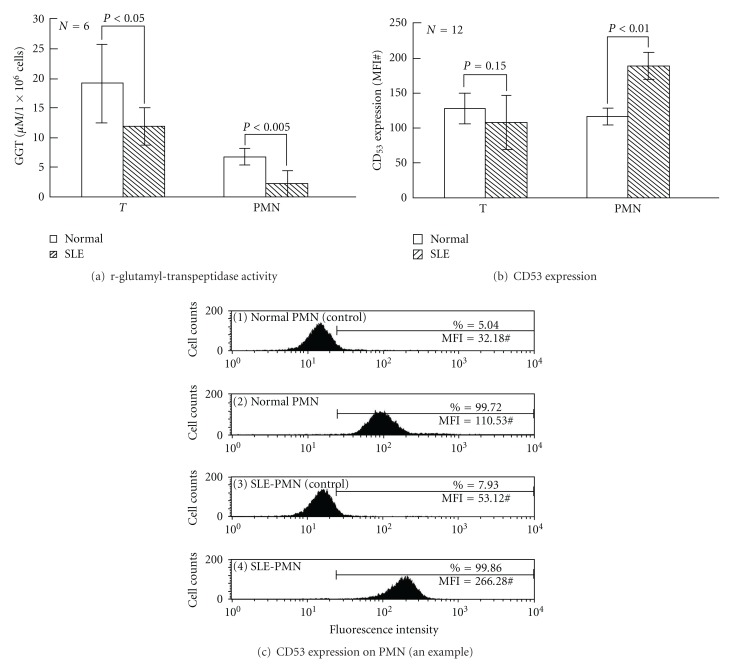
Comparison of *γ*-glutamyl-transpeptidase (GGT) enzyme activity and CD53 expression in T and PMN of normal and SLE patients. (a) GGT enzyme activity. (b) Surface CD53 expression. (c) A typical case demonstrating surface CD53 expression on normal and SLE-PMN using flow cytometry. A similar tendency was seen in the cells of an additional 3 normal individuals and 3 SLE patients.

Because GGT is crucial for enhanced antioxidant capacity through the recapture of glutathione molecules decreased intracellular GSH levels in SLE-T and SLE-PMN may be due to reduced GGT activity in the cells. GTT activity was below normal in both T and PMN from active SLE patients (Figure 6(a)). However, CD53 expression in SLE-T was not different from normal T (Figure 6(b), left panel). Unexpectedly, the CD53 expression in SLE-PMN was higher than in normal PMN (Figure 6(b), right panel). A representative case is shown in Figure 6(c). This may suggest that a compensatory mechanism is activated to limit oxidative damage in SLE-PMN [2, 4, 31] that results from the GSH-Px defect of the cells.

## 4. Discussion

T lymphocytes and PMN are very efficient cells for body defense. These cells need constant energy for basic housekeeping and specific actions against infections. Oxidative stress is increased in patients with active SLE [1–4]. Shah et al. [32] further demonstrated that the increased oxidative stress in SLE is related to Th1 cytokine IFN-*γ* and IL-12 and to disease activity. However, there have been no reports detailing the molecular basis of increased oxidative stress in active SLE. In the present study, we observed several interesting abnormalities of cellular bioenergetics and redox capacity in SLE-T and SLE-PMN. These include (a) increased intracellular basal lactate levels and decreased ATP production, (b) decreased glucose-uptake by these cells attributable to the defective expression of the glucose transporters, GLUT-3 and GLUT-6, (c) reduced redox capacity, including decreased intracellular GSH levels and enzymatic activity of GSH-Px and GGT, and (d) normal CD53 expression in SLE-T but increased expression in SLE-PMN. In addition, some other important contributory factors such as mitochondrial hyperpolarization, immune-mediated systemic tissue inflammation/damage, or accelerated atherosclerosis-mediated tissue hypoxia, may also be involved in the increased oxidative stress that occurs in patients with active SLE.

Glucose is a required energy source for many cells, particularly those in the immune system. Glucose is needed for oxidative and nonoxidative ATP production, anaerobic production of various sugar-containing macromolecules, and cell proliferation [6, 27–29]. Tan et al. [33] and Schuster et al. [34] demonstrated that after activation, PMN is critically dependent on glucose uptake and glycolysis for supplying the necessary energy to conduct effector functions. The bacterial products GM-CSF and phorbol myristate acetate enhance glucose uptake by sequential activation of neutrophilic protein tyrosine kinase C [32], p38 MAPK, and hypoxia-inducible factor pathways [34]. Fu et al. [13] further demonstrated that GLUT-1 and GLUT-3 expression provides cellular fuel for immune responses. Maciver et al. [29] showed that if glucose uptake is limited, glycolytic flux decreases to a level that no longer sustains viability, and the proapoptotic Bcl-2 family becomes activated, promoting cell death. This may lead to increased glycolytic capacity and a high rate of lactate formation from glucose even under aerobic conditions [27]. Our finding of decreased glucose uptake in SLE immune cells may be reflected in an increase of basal lactate levels and cell apoptosis in the patients. On the other hand, ATP generation is mainly derived from glucose metabolism via glycolysis or oxidative phosphorylation [35]. The elevated basal lactate levels observed in SLE PMN suggest an anaerobic metabolic state in these activated cells. In addition, compared to normal cells, SLE-T and SLE-PMN tend to have decreased ATP production (Figure 1(d)). This decreased ATP production reflects mitochondrial functions impaired by activation-induced cell death [36, 37] and decreased redox capacity in SLE [2, 4, 30]. Abnormal glucose bioenergetics in SLE cells owing to defective expression of GLUT-3 and GLUT-6 on the cell surface can lead to impaired immune functions in active SLE. 

Proinflammatory cytokines induce a variety of metabolic changes in the utilization of carbohydrates and fat [38]. IL-1*β*, TNF-*α*, and LPS effectively facilitate glucose uptake and modulate the expression of different glucose transporters in experimental cells [38–40]. SLE serum is thought to contain high levels of different Proinflammatory cytokines including TNF-*α*, IL-6, IL-12, IFN-*α*, and IFN-*γ*  that  may change glucose transporter expression and glucose metabolism [40–43]. We speculated that alterations in the intracellular bioenergetics of SLE immune cells are the result of long-term cell activation, which leads to production of a number of Proinflammatory cytokines. In clinical settings, abnormal redox states in the body fluid and blood cells of patients with some diseases, such as rheumatoid arthritis [44, 45], cardiovascular disorders [46, 47], and atopic asthma [48] have been reported. In the present study, we demonstrated that SLE-T and SLE-PMN have impaired intracellular redox capacity and increased oxidative damage. The defective redox capacity is due to SLE disease activity *per se* rather than the effects of immunosuppressive therapy. However, it is worth noting that any differences between the GSH levels of T and PMN in SLE patients and healthy individuals may be relevant to cell functions, but is unlikely to be relevant to the total ROS load. This is because any such differences would be masked by the large antioxidant capacity of plasma due to GSH, catalase, SOD, and numerous other free oxygen radical scavengers. The manipulation of cell redox states may become an alternative strategy for improving immune responses in some forms of cancer [17] and immune hyporesponsiveness states. Furthermore, Maurice et al. [44] demonstrated that an altered redox state is responsible for the hyporesponsiveness of rheumatoid synovial T cells. Supplementation of GSH with the glutathione precursor, *N*-acetyl-l-cysteine, enhances mitogen-induced proliferative responses and IL-2 production of synovial T lymphocytes. However, patients with atopic asthma [48], ischemic heart disease [46], and stroke [47] exhibit reduced redox capacity, but do not show distinct immune hyporesponsiveness to external stimuli. Wahl et al. [49] noted that chronically activated T cells such as SLE-T rely primarily on oxidative metabolism for ATP synthesis, suggesting that chronic antigen stimulation may be the basis for the metabolic abnormalities seen in SLE patients. Whether immune hyporesponsiveness in active SLE can be restored by supplementation with glutathione or its precursors is now under investigation. It would be interesting to directly measure the ROS generation in T and PMN and correlate it with autophagic activity, apoptosis, and other cellular functions in active SLE patients. 

In conclusion, we found that impaired glucose bioenergetics and redox capacity in SLE-T and PMN are related to impaired cellular immune function and increased oxidative stress in active SLE. 

## Figures and Tables

**Table 1:** The demographic features and clinical data of normal, active, and inactive SLE groups.

Parameters	Normal (*N* = 52)	Active SLE (*N* = 42)	Inactive SLE (*N* = 12)
Age, years (mean ± S.D.)	27.3 ± 8.7	25.7 ± 10.4	25.6 ± 9.4
Gender (female : male)	47 : 5	50 : 4	11 : 1
Disease duration	—	3–9 years	4–8 years
SLEDAI score	—	6–13	0–5
Serum C3 (mg/dL)	91.1 ± 8.5	50.7 ± 11.1	87.4 ± 13.5
Serum C4 (mg/dL)	19.4 ± 2.4	8.8 ± 3.2	16.7 ± 2.9
Anti-dsDNA(IU/mL)	—	259.4 ± 30.7	107.7 ± 13.8
Medications			
Prednisolone	—	17.6 ± 10.7 mg/D	5.3 ± 6.2 mg/D
Azathioprine	—	50–100 mg/D	—
Hydroxychloroquine	—	400 mg/D	200–400 mg/D

## References

[1] Avalos I, Chung CP, Oeser A (2007). Oxidative stress in systemic lupus erythematosus: relationship to disease activity and symptoms. *Lupus*.

[2] Wang G, Pierangeli SS, Papalardo E, Ansari GAS, Khan MF (2010). Markers of oxidative and nitrosative stress in systemic lupus erythematosus. *Arthritis and Rheumatism*.

[3] Gergely P, Grossman C, Niland B (2002). Mitochondrial hyperpolarization and ATP depletion in patients with systemic lupus erythematosus. *Arthritis and Rheumatism*.

[4] Kurien BT, Scofield RH (2003). Free radical mediated peroxidative damage in systemic lupus erythematosus. *Life Sciences*.

[5] Buttgereit F, Burmester GR, Brand MD (2000). Bioenergetics of immune functions: fundamental and therapeutic aspects. *Immunology Today*.

[6] Krauss S, Brand MD, Buttgereit F (2001). Signaling takes a breath—New quantitative perspectives on bioenergetics and signal transduction. *Immunity*.

[7] Van Schaftingen E, Gerin I (2002). The glucose-6-phosphatase system. *Biochemical Journal*.

[8] Bell GI, Kayano T, Buse JB (1990). Molecular biology of mammalian glucose transporters. *Diabetes Care*.

[9] Burant CF, Bell GI (1992). Mammalian facilitative glucose transporters: evidence for similar substrate recognition sites in functionally monomeric proteins. *Biochemistry*.

[10] Fukumoto H, Seino S, Imura H, Seino Y, Bell GI (1988). Characterization and expression of human HepG2/erythrocyte glucose-transporter gene. *Diabetes*.

[11] Estrada DE, Elliott E, Zinman B (1994). Regulation of glucose transport and expression of GLUT3 transporters in human circulating mononuclear cells: studies in cells from insulin-dependent diabetic and nondiabetic individuals. *Metabolism*.

[12] Joost HG, Thorens B (2001). The extended GLUT-family of sugar/polyol transport facilitators: nomenclature, sequence characteristics, and potential function of its novel members. *Molecular Membrane Biology*.

[13] Fu Y, Maianu L, Melbert BR, Garvey WT (2004). Facilitative glucose transporter gene expression in human lymphocytes, monocytes, and macrophages: a role for GLUT isoforms 1, 3, and 5 in the immune response and foam cell formation. *Blood Cells, Molecules, and Diseases*.

[14] Viora M, Quaranta MG, Straface E, Vari R, Masella R, Malorni W (2001). Redox imbalance and immune functions: opposite effects of oxidized low-density lipoproteins and *N*-acetylcysteine. *Immunology*.

[15] Holmgren A (1989). Thioredoxin and glutaredoxin systems. *Journal of Biological Chemistry*.

[16] Holmgren A (1979). Glutathione-dependent synthesis of deoxyribonucleotides. Characterization of the enzymatic mechanism of *Escherichia coli* glutaredoxin. *Journal of Biological Chemistry*.

[17] Sen CK (1997). Nutritional biochemistry of cellular glutathione. *Journal of Nutritional Biochemistry*.

[18] Carlisle ML, King MR, Karp DR (2003). *γ*-Glutamyl transpeptidase activity alters the T cell response to oxidative stress and Fas-induced apoptosis. *International Immunology*.

[19] Pedersen-Lane JH, Zurier RB, Lawrence DA (2007). Analysis of the thiol status of peripheral blood leukocytes in rheumatoid arthritis patients. *Journal of Leukocyte Biology*.

[20] Gladman DD, Ibanez D, Urowitz MB (2007). Systemic lupus erythematosus disease activity index 2000. *Journal of Rheumatology*.

[21] Li KJ, Lu MC, Hsieh SC (2006). Release of surface-expressed lactoferrin from polymorphonuclear neutrophils after contact with CD4+T cells and its modulation on Th1/Th2 cytokine production. *Journal of Leukocyte Biology*.

[22] Frauwirth KA, Riley JL, Harris MH (2002). The CD28 signaling pathway regulates glucose metabolism. *Immunity*.

[23] Shikhman AR, Brinson DC, Valbracht J, Lotz MK (2001). Cytokine regulation of facilitated glucose transport in human articular chondrocytes. *Journal of Immunology*.

[24] Kato Y, Mori Y, Makino Y (1999). Formation of N(*ε*)-(hexanonyl)lysine in protein exposed to lipid hydroperoxide. A plausible marker for lipid hydroperoxide. *Journal of Biological Chemistry*.

[25] Loft S, Fischer-Nielsen A, Jeding IB, Vistisen K, Enghusen Poulsen H (1993). 8-Hydroxydeoxyguanosine as a urinary biomarker of oxidative DNA damage. *Journal of Toxicology and Environmental Health*.

[26] Tsokos GC, Liossis SNC (1999). Immune cell signaling defects in lupus: activation, anergy and death. *Immunology Today*.

[27] Greiner EF, Guppy M, Brand K (1994). Glucose is essential for proliferation and the glycolytic enzyme induction that provokes a transition to glycolytic energy production. *Journal of Biological Chemistry*.

[28] Frauwirth KA, Thompson CB (2004). Regulation of T lymphocyte metabolism. *Journal of Immunology*.

[29] Maciver NJ, Jacobs SR, Wieman HL, Wofford JA, Coloff JL, Rathmell JC (2008). Glucose metabolism in lymphocytes is a regulated process with significant effects on immune cell function and survival. *Journal of Leukocyte Biology*.

[30] Misso NLA, Peroni DJ, Neil Watkins D, Stewart GA, Thompson PJ (1998). Glutathione peroxidase activity and mRNA expression in eosinophils and neutrophils of asthmatic and non-asthmatic subjects. *Journal of Leukocyte Biology*.

[31] Morgan PE, Sturgess AD, Davies MJ (2005). Increased levels of serum protein oxidation and correlation with disease activity in systemic lupus erythematosus. *Arthritis and Rheumatism*.

[32] Shah D, Kiran R, Wanchu A, Bhatnagar A (2010). Oxidative stress in systemic lupus erythematosus: relationship to Th1 cytokine and disease activity. *Immunology Letters*.

[33] Tan AS, Ahmed N, Berridge MV (1998). Acute regulation of glucose transport after activation of human peripheral blood neutrophils by phorbol myristate acetate, fMLP, and granulocyte-macrophage colony-stimulating factor. *Blood*.

[34] Schuster DP, Brody SL, Zhou Z (2007). Regulation of lipopolysaccharide-induced increases in neutrophil glucose uptake. *American Journal of Physiology *.

[35] Fox CJ, Hammerman PS, Thompson CB (2005). Fuel feeds function: energy metabolism and the T-cell response. *Nature Reviews Immunology*.

[36] Hsieh SC, Sun KH, Tsai CY (2001). Monoclonal anti-double stranded DNA antibody is a leucocyte-binding protein to up-regulate interleukin-8 gene expression and elicit apoptosis of normal human polymorphonuclear neutrophils. *Rheumatology*.

[37] Hsieh SC, Yu HS, Lin WW (2003). Anti-SSB/La is one of the antineutrophil autoantibodies responsible for neutropenia and functional impairment of polymorphonuclear neutrophils in patients with systemic lupus erythematosus. *Clinical and Experimental Immunology*.

[38] Garcia-Welsh A, Schneiderman JS, Baly DL (1990). Interleukin-1 stimulates glucose transport in rat adipose cells. Evidence for receptor discrimination between IL-1*β* and IL-1*α*. *FEBS Letters*.

[39] Hernvann A, Aussel C, Cynober L, Moatti N, Ekindjian OG (1992). IL-1*β*, a strong mediator for glucose uptake by rheumatoid and non-rheumatoid cultured human synoviocytes. *FEBS Letters*.

[40] Bedard S, Marcotte B, Marette A (1997). Cytokines modulate glucose transport in skeletal muscle by inducing the expression of inducible nitric oxide synthase. *Biochemical Journal*.

[41] Kim T, Kanayama Y, Negoro N, Okamura M, Takeda T, Inoue T (1987). Serum levels of interferons in patients with systemic lupus erythematosus. *Clinical and Experimental Immunology*.

[42] Grondal G, Gunnarsson I, Ronnelid J, Rogberg S, Klareskog L, Lundberg I (2000). Cytokine production, serum levels and disease activity in systemic lupus erythematosus. *Clinical and Experimental Rheumatology*.

[43] Sabry A, sheashaa H, El-husseini A (2006). Proinflammatory cytokines (TNF-*α* and IL-6) in Egyptian patients with SLE: its correlation with disease activity. *Cytokine*.

[44] Maurice MM, Nakamura H, Van Der Voort EAM (1997). Evidence for the role of an altered redox state in hyporesponsiveness of synovial T cells in rheumatoid arthritis. *Journal of Immunology*.

[45] Bazzichi L, Ciompi ML, Betti L (2002). Impaired gluthathione reductase activity and levels of collagenase and elastase in synovial fluid in rheumatoid arthritis. *Clinical and Experimental Rheumatology*.

[46] Porter M, Pearson DJ, Suarez-Mendez VJ, Blann AD (1992). Plasma, platelet and erythrocyte glutathione peroxidases as risk factors in ischaemic heart disease in man. *Clinical Science*.

[47] Ishibashi N, Prokopenko O, Reuhl KR, Mirochnitchenko O (2002). Inflammatory response and glutathione peroxidase in a model of stroke. *Journal of Immunology*.

[48] Comhair SAA, Bhathena PR, Farver C, Thunnissen FBJM, Erzurum SC (2001). Extracellular glutathione peroxidase induction in asthmatic lungs: evidence for redox regulation of expression in human airway epithelial cells. *FASEB Journal*.

[49] Wahl DR, Petersen B, Warner R, Richardson BC, Glick GD, Opipari AW (2010). Characterization of the metabolic phenotype of chronically activated lymphocytes. *Lupus*.

